# Effects of exposure to lisdexamfetamine dimesylate on hepatic parameters of pubertal rats

**DOI:** 10.1007/s00210-026-05370-1

**Published:** 2026-05-06

**Authors:** João Vinícius Honório da Silva, Letícia Cavalcante Santos, Rafaela Pires Erthal, Dayane Priscila dos Santos, Camila Rodrigues Ferraz, Camila Franciele de Souza, Luís Eduardo Duarte Gonçalves, Waldiceu Aparecido Verri, Niels Olsen Saraiva Câmara, Ernane Torres Uchôa, Glaura Scantamburlo Alves Fernandes, Fábio Goulart de Andrade

**Affiliations:** 1 Department of General Biology, Biological Sciences Center, State University of Londrina – UEL, Londrina, Paraná Brazil; 2 Department of Pathology, Biological Sciences Center, State University of Londrina – UEL, Londrina, Paraná Brazil; 3 Department of Histology, Biological Sciences Center, State University of Londrina – UEL, Londrina, Paraná Brazil; 4 Department of Physiological Sciences, Biological Sciences Center, State University of Londrina – UEL, Londrina, Paraná Brazil; 5 Department of Immunology, Biomedical Sciences Institute, University of São Paulo – USP, São Paulo, Brazil

**Keywords:** Amphetamine, Hepatotoxicity, Inflammation, Injury

## Abstract

Lisdexamfetamine dimesylate (LDX) is a psychostimulant, that has been widely recommended in recent years for the treatment of Attention-Deficit/Hyperactivity Disorder. The present study aims to evaluate the effects of LDX administration on the hepatic morphophysiology of pubertal Wistar rats, since the liver plays a central role in systemic metabolism. Male Wistar rats were randomly distributed into two experimental groups: LDX group (LDX) — received 11.3 mg/kg/day of LDX diluted in tap water; and the Control group (C) — received tap water only. Animals were treated by gavage during puberty, from postnatal day (PND) 25 to PND 65. The results showed that administration of LDX decreased white adipose tissue weight without altering overall body weight gain. Plasma biochemical analysis demonstrated an increase in plasma total cholesterol and in the concentration of hepatic transaminases in LDX rats. In addition, LDX animals exhibited an increase in N-acetyl-β-D-glucosaminidase activity, indirectly indicating macrophage recruitment to the liver. This study shows that daily exposure to LDX during puberty causes early liver inflammation and damage due, in part, to increased hepatic transaminases and associated with indirect signs of macrophage recruitment. This finding highlights the importance of monitoring the indiscriminate use of amphetamines and alerting patients to possible liver disorders.

**Graphical Abstract:**

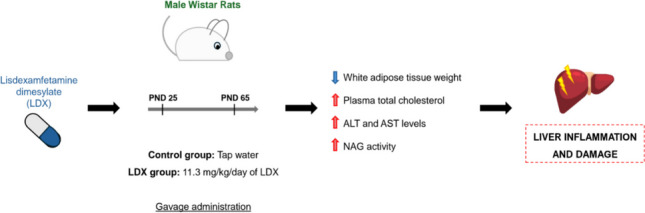

## Introduction

Amphetamine, a synthetic central nervous system stimulant, has been commercially available since the 1930s (Heal et al. 2013). Its primary mechanism of action involves the inhibition of dopamine and norepinephrine transporters, leading to elevated levels of these catecholamines within the synaptic cleft (Faraone 2018). Physiologically, dopamine facilitates cognitive processes and motor activities (Faraone et al. 2015), while serotonin is involved in modulating the dopaminergic system and emotional responses (Dunn et al. 2019). The first report regarding the effectiveness of amphetamine was published by Bradley (1937), who used it in children with behavior problems. As a result of further scientific study, amphetamines and their derivatives began to be used for several disorders, including Attention-Deficit/Hyperactivity Disorder (ADHD) (Connolly et al. 2015). As the most common neurodevelopmental disorder, ADHD is characterized by persistent symptoms of inattention, hyperactivity, and impulsivity (American Psychiatric Association, 2013). The estimated worldwide prevalence of ADHD in children and adolescents is 5.29%, with a higher incidence in men (Polanczyk et al. 2007).

One of the amphetamines widely recommended to treat ADHD is lisdexamfetamine dimesylate (LDX), an inactive prodrug of dextroamphetamine (D-AMPH) (Comiran et al. 2016). According to Goodman (2010), LDX is the first long-acting psychostimulant prodrug intended for the treatment of ADHD, consisting of a D-AMPH molecule covalently linked to the essential amino acid L-lysine. LDX is mainly sold in capsules, meaning its primary route of administration is oral (Steer et al. 2012; Ermer et al. 2016). This drug is mainly absorbed via peptide transporter 1 (PEPT1) (Ermer et al. 2012), which is abundantly expressed in the gut (Viennois et al. 2018). Upon absorption, LDX reaches the portal bloodstream, where it is quickly enzymatically hydrolyzed (Ermer et al. 2016). This metabolism occurs in plasma and is carried out in the cytosol of erythrocytes by an unknown aminopeptidase (Sharman and Pennick 2014). Najib et al. (2020) reported that D-AMPH crosses the blood–brain barrier, reaching the central nervous system, and acts by increasing noradrenergic and dopaminergic neurotransmission. Its metabolites are subsequently eliminated by renal excretion (Krishnan et al. 2008).

There is substantial clinical and experimental evidence (Montiel-Duarte et al. 2002; El-Tawil et al. 2011; Dias Da Silva et al. 2013) as well as case reports (Hood and Nowicki 2010; Vanga et al. 2013) demonstrating hepatotoxicity caused by exposure to amphetamines in general; however, there is little information about LDX at this dosage. It is noteworthy that although LDX is used during the developmental phase in humans, there are no studies on LDX hepatotoxicity during this phase, i.e., puberty. Thus, as LDX is a relatively new drug and has a different pharmacological profile compared to other amphetamines (Heal et al. 2013), an extensive evaluation of its toxicological profile of LDX is necessary in different organs and tissues, to assess possible alterations in inflammatory parameters, oxidative status and morphological parameters. Based on this context and an extensive review of the current scientific literature, the aim of the present study was to evaluate whether the administration of LDX causes damage to the liver morphophysiology of pubertal Wistar rats.

## Material and methods

### Animals and experimental conditions

Twenty male Wistar rats, aged postnatal day 22 (PND 22), were obtained from the Central Animal House of the Londrina State University (CCB—UEL), Paraná, Brazil. These animals were acclimated to the new environment (at the Animal House of the Laboratory of Toxicology and Metabolic Dysfunction of Reproduction) for 3 days before the beginning of the experiment. During the experimental period (PND 25 to 65), the rats were housed in polypropylene cages (5 rats per cage) with laboratory-grade pine shavings as bedding. The animals were kept under controlled light conditions (12 h light/dark cycle, with lights on at 07:00 am), maintained at a constant temperature (23 ± 2 °C), and had access to standard commercial laboratory chow and tap water *ad libitum* throughout the experimental period. The body weight of all rats was measured three times a week and expressed in grams (g). All animal care and handling procedures were conducted in accordance with Brazilian National Council for the Control of Animal Experimentation (CONCEA) guidelines and Brazilian legislation (No. 11,794/2008). The research was approved by the Ethics Committee on Animal Use of Londrina State University (OF. CIRC. CEUA N° 82/2019 and addendum OF. CIRC. CEUA N° 004/2021). All experimental procedures were performed by the same evaluators.

### LDX exposure and experimental design

The animals were randomly assigned to two experimental groups of 10 animals each: control (C) and lisdexamfetamine (LDX). The animals in the LDX group received lisdexamfetamine dimesylate by gavage of 11.3 mg/kg b.w. (body weight) diluted in tap water. The calculation was performed using the conversion of animal doses (rat) to human-equivalent doses based on body surface area (Food and Drug Administration 2013). This dose was selected considering that it is equivalent to administering a 70 mg capsule of the drug and corresponds to 1.13% of the oral LD50 for rats (oral LD50 for rats > 1000 mg/kg) (Krishnan and Montcrief 2007). The control group received only the vehicle (tap water). All groups were treated daily, by oral gavage, for 40 consecutive days (between PND 25 and 65). This period corresponds to the juvenile and peripubertal phase of Wistar rats (Ojeda et al. 1980), which is a similar life stage to that recommended for human use of LDX, in childhood and puberty (Najib et al. 2020).

### Preparation of lisdexamfetamine dimesylate solution

Lisdexamfetamine dimesylate (Venvanse®, Patheon Pharmaceuticals Inc., Ohio, USA) was diluted daily in the corresponding volume of tap water.

### Blood and liver collection

On the 40th day of the experiment (PND 65), rats were subjected to an overnight fast with free access to water. Blood glucose levels were determined from a tail vein blood drop using Accu-Chek Active strips (Ref. 07124112001. Roche, Taquara, RJ, Brazil).

On PND 66, the rats were intraperitoneally anesthetized with a combination of ketamine 75 mg/kg b.w. (Sedomin® 10%, Avellaneda, Argentina) and xylazine 10 mg/kg b.w. (Anasedan®, Paulínia, Brazil), weighed and euthanized by inferior vena cava puncture. Blood samples were collected in the presence of heparin (Hemofol®, São Paulo, Brazil) and plasma was stored at − 20 °C for determination of plasma aspartate aminotransferase (AST), alanine aminotransferase (ALT), cholesterol, triglycerides, and free fatty acid concentrations (*n* = 10 rats per group).

The liver and white adipose tissue (retroperitoneal, perigonadal, and perirenal) were removed, and their absolute and relative weights were determined (*n* = 10 per group). Relative weight was calculated as the ratio of organ weight to animal body weight multiplied by 100.

The largest lobe of the liver (large right middle lobe) was selected and divided into 4 parts for each analysis: histological and histochemical analysis (*n* = 6 per group); inflammatory profile (*n* = 10 per group); gene expression (*n* = 5 per group); and redox balance status assay (*n* = 10 per group).

### Biochemical analyses

Plasma levels of total cholesterol, triglycerides, and hepatic transaminases (AST and ALT) were measured using commercially available kits (Laborclin and Labtest Diagnóstica SA, Brazil, respectively) according to the manufacturers’ instructions. Plasma free fatty acid concentrations were determined spectrophotometrically at 550 nm, following the extraction and quantification protocols described by de Souza et al. (2019). All samples were measured in duplicate.

### Processing and morphometric and histological evaluation

Liver samples were fixed in aqueous Bouin for 48 h. The samples were dehydrated through graded ethanol baths to ensure complete removal of water and prevent tissue shrinkage (started with 70% ethanol for 30 min, followed by three baths of 95% ethanol, 30 min each, and then three consecutive baths of 100% ethanol, 30 min each), and subsequently cleared in four xylene baths, 30 min each. Samples were then infiltrated with molten paraffin through four consecutive paraffin baths, 30 min each. After infiltration, tissues were embedded in paraffin using molds and allowed to cool completely.

The samples were sectioned at 7 µm (2 non-serial sections per animal). The thickness used here (7 µm), optimized for collagen polarization analysis, may differ from the standard 3–4 µm used for routine morphological evaluation. The sections were stained with hematoxylin and eosin (HE) and evaluated in a Moticam® image capture system coupled to a photomicroscope (Motic, Xiamen, China) and analyzed using Motic Image Plus 3.0® software.

H&E staining was performed to evaluate hepatic morphology in all samples. Paraffin sections were deparaffinized in two xylene baths, 10 min each, followed by rehydration in 100%, 95%, and 70% ethanol (5 min each) and distilled water (5 min). Slides were stained with hematoxylin for 1 min and washed in running tap water for 5 min. Sections were then counterstained with eosin for 2 min and briefly rinsed in distilled water, followed by rapid passages through 70% and 95% ethanol. Dehydration continued in 95% ethanol (5 min) and two baths of 100% ethanol (10 min each). Slides were cleared in alcohol–xylene (1:1, 10 min) and 2 xylene baths (10 min each), then mounted with Canada balsam.

For measurement of morphometric parameters, 20 photomicrographs of each animal were obtained at 400 × magnification: 10 images of the portal regions and 10 of the central regions. The average diameter of the portal vein, hepatic artery, bile duct, and central vein was measured. In each image, the diameter of 5 sinusoids next to the veins was also measured, as well as the count of leukocytes adhered to the vessel lumen.

Interpretation of the histological alterations was based on previous studies (Samelo et al. 2020; Kaya Tektemur et al. 2021). The following parameters of liver damage were evaluated: necrosis, hydropic degeneration, foci of defense cells, steatosis, vascular congestion, and sinusoidal congestion. The frequency of each parameter of liver injury was observed and scored as absent (0), mild (1), moderate (2), and severe (3).

### Histochemical analyses

#### Azan Heidenhain trichrome stain

The slides (with 2 non-serial sections per animal) were first deparaffinized in two baths of xylene (5–7 min), rehydrated through graded ethanol solutions (100%, 90%, 70%) (5 min each) and rapidly rinsed in distilled water. The dye used was Azo-Carmin G (20 min), washed thoroughly, and destained in 70% ethanol (5 min). After rinsing, sections were incubated in phosphotungstic acid as a catalyst (25 min), washed again, and stained with Aniline blue for 20 min. Excess stain was removed with distilled water, and sections were dehydrated through 70%, 90%, and 100% ethanol (5 min each), and cleared in two baths of xylene (5 min each).

Using the Moticam® image capture system coupled to a photomicroscope (Motic, Xiamen, China), at 400 × magnification, 5 images from each region (central and portal) were captured per animal. Using Image-Pro PLUS® software version 4.5, the blue pixels, which indicate collagen in each image were quantified (Taatjes et al. 2019; Honório da Silva et al. 2024). The data were compared to define the distribution of these tissue components between the regions of the groups.

The regions close to the vessels were chosen for evaluation in the histochemical analyses, because of the hemodynamic and functional scheme of the liver. As there is a difference in the blood that leaves the portal triad and reaches the central vein, the functions of hepatocytes will also differ according to their location in the hepatic lobe (Trefts et al. 2017; Freitas-Lopes et al. 2017). For this reason, the lobe is divided into 3 metabolic zones (Grijalva and Vakili 2013).

### Picrosirius-polarization method

Picrosirius red is a histochemical technique used to differentiate between mature (type I) and newly synthesized (type III) collagen fibers when observed under polarized light, making it possible to determine the percentage of each type of collagen in tissues (Charan Gowda et al. 2017). In short, the slides (with 2 non-serial sections per animal) were deparaffinized in two baths of xylene (10 min each) and rehydrated through 100%, 95%, 70%, and 80% ethanol (5 min each), followed by distilled water (5 min). Samples were immersed in Picrosirius solution (0.2 g of Sirius red in 200 mL of saturated picric acid) for 1 h and rinsed in distilled water and running water for 5 min. Slides were then dehydrated in 90% ethanol, 100% ethanol, alcohol-xylene (1:1) (5 min each), and four xylene baths (10 min each). Using the Moticam® image capture system coupled to a polarized light microscope (Motic® BA410), at 400 × magnification, 5 images from each region (central and portal) were captured per animal. Image-Pro PLUS® software version 4.5 was used to quantify the red pixels for type I collagen fibers and green pixels for type III collagen fibers (Pupim et al. 2017). The data were compared to define the distribution of these tissue components between the regions of the groups.

### Toluidine blue stain

This stain is mainly used to stain the metachromatic granules of mast cells and thus enables the quantification of the mast cells in tissues (Ribatti 2018). The tissue sections were stained with 1% toluidine blue to identify mast cells, which were classified with the aid of a light microscope into intact and degranulated forms. The analysis of the count of these cells was performed by analyzing the entire length of the sections. (2 non-serial sections per animal) using a light microscope and quantifying and classifying each observed mast cell (Paula Franco Punhagui et al. 2016).

### Inflammatory profile

#### Myeloperoxidase activity

Myeloperoxidase (MPO) activity is a method of indirect assessment of neutrophil recruitment, since MPO is an abundant enzyme in the azurophilic granules of neutrophils. This assay was performed as previously described by Ferraz et al. (2021). Liver samples of approximately 100 mg were removed and stored in a cryotube with 200 µL of 50 mM K_2_PO_4_ buffer solution (pH 6.0), composed of hexadecyltrimethylammonium bromide (HTAB; 0.5%), at − 80 °C. On the day of the assay, these samples were homogenized on ice using a Tissue-Tearor (Biospec®). Subsequently, the homogenates were centrifuged (600 g, 2 min, 4 °C), and the resulting supernatants (15 µL) were blended with 200 µL of 50 mM K_2_PO_4_ buffer, pH 6.0, containing 0.167 mg/mL of o-dianisidine dihydrochloride and 0.015% hydrogen peroxide. The absorbance of MPO activity was determined spectrophotometrically at 450 nm (Multiskan GO Microplate, Thermo Fischer Scientific, Vantaa, Finland). The results of the MPO activity assay are expressed as the number of neutrophils per mg of protein using a neutrophil standard curve (196–400,000 neutrophils).

### N-acetyl-β-D-glucosaminidase activity

N-acetyl-β-D-glucosaminidase (NAG) activity is a colorimetric method to assess macrophage recruitment. This assay was performed as previously described by Ferraz et al. (2021). Firstly, 10 µL of the supernatant obtained from the MPO activity procedure was separated and added to a 96-well plate, followed by the addition of 40 µL of 50 mM phosphate buffer, pH 6.0. The reaction was initiated by the addition of 2.24 mM 4-nitrophenyl NAG. The plate was incubated at 37 °C for 10 min, and the reaction was stopped by the addition of 100 µL of 0.2 M glycine buffer, pH 10.6. NAG enzymatic activity was determined spectrophotometrically at 400 nm (Multiskan GO Microplate, Thermo Fischer Scientific, Vantaa, Finland). The results of the NAG activity are expressed as the number of macrophages per mg of protein using a standard curve of macrophages (196–400,000 macrophages).

### Oxidative status assay

Liver samples were homogenized in PBS (pH 7.4) using an Ultra-turrax (IKA Works, Wilmington, USA), and centrifuged (500 g, 10 min). The supernatant was used to determine the protein concentration by the Bradford method (Bradford 1976), and all samples were subsequently normalized to 1 mg/mL protein. Lipid peroxidation was measured by measuring thiobarbituric acid reactive substances (TBARS), expressed as malondialdehyde (MDA) equivalents (Buege and Aust 1978). Catalase (CAT) activity was determined by monitoring H_2_O_2_ consumption at 240 nm (Aebi 1984). Glutathione S-transferase (GST) activity was assessed by measuring the conjugation of GSH with CDNB at 340 nm (Keen et al. 1976). Reduced glutathione (GSH) levels were quantified using the enzymatic recycling method with CDNB at 412 nm (Rahman et al. 2006). Superoxide dismutase (SOD) activity was evaluated by the inhibition of nitroblue tetrazolium (NBT) reduction at 560 nm (Senthilkumar et al. 2021). All biochemical oxidative assays were adapted for microplate reading, measured in duplicate, and corrected for protein content (Honório da Silva et al. 2024).

### RNA extraction, cDNA synthesis and RT‐qPCR

Total RNA was isolated from the liver samples using TRIzol reagent (Cat. 15,596,018, Invitrogen, Waltham, MA, USA) according to the manufacturer’s instructions. cDNA was synthesized from 2000 ng of RNA using the M‐MLV Reverse Transcriptase System (Promega, USA). The qPCR reaction was performed with 0.5 µL (500 nM) of the forward primer, 0.5 µL (500 nM) of the reverse primer, 1 µL of purified water, 5 µL of SYBR Green Master Mix (Cat. 4,309,155, Applied Biosystems, Waltham, MA, USA), and 4 µL of the previously diluted cDNA. All reactions were performed on the QuantStudio 12 K Flex Real‐Time PCR System device (Applied Biosystems, USA). All samples were analyzed in technical triplicates, and the GAPDH gene was chosen as housekeeping. Quantification was performed by the 2^−ΔΔCT^ method. We used the following primer sequences: *Gapdh* (F: AGGTCGGTCTGAACGGATTTG; R: TGTAGACCATGTAGTTGAGGTCA); *Cd206* (F: TCTGTGCCTATCTCTCCAACCA; R: CGACTTCAATTTCATAAGGGCATA); *Il10* (F: CCAAGCCTTGTCAGAAATGATCA; R: CAGCTTCTCTCCCAGGGAATTC); *Tnf-α* (F: CAGACCCTCACACTCAGATCA; R: CTCCGCTTGGTGGTTTGCTA); and *Tgf-β* (F: AACTATTCGTTCAGCTCCACAGAGA; R: AAGTTGGCATGGTAGCCCTT).

### Statistical analysis

A formal a priori power analysis was not performed. Sample sizes were determined based on previous studies using similar experimental designs and were considered sufficient to detect biologically relevant differences in the evaluated outcomes.

The parameters were submitted to the Shapiro–Wilk test to verify the normal distribution of the data and classify them into parametric and non-parametric data. The homogeneity of the variance between the groups was assessed by the Levene test. Comparisons of data between two groups were performed using the unpaired two-tailed Student’s *t*-test or the Mann–Whitney test. Data are presented as mean ± S.E.M. (standard error of the mean), with *P* < 0.05 was considered statistically significant. The statistical analyses and graph design for the results were performed using GraphPad Prism for Windows version 8.2.1 (San Diego, CA, USA).

## Results

### Body and organ weights

The body weight (gain, initial, and final) and relative and absolute organ weights (liver and white adipose tissue) are shown in Table 1. There were no significant differences between groups for body weight parameters. However, a reduction was observed in the weight of the white adipose tissue (absolute and relative weight) in animals in the LDX group compared with the Control group.

**Table 1:** Body and organ weight

Parameters		Control group (C)	LDX group (LDX)	*P *value
*Absolute weight (g)*				
	Initial	61.6 ± 1.1	62.9 ± 1.7	0.5361
	Final	272.0 ± 4.9	256.0 ± 8.4	0.1204
	Weight gain	211.0 ± 4.2	193.0 ± 7.1	0.0531
	Liver	8.81 ± 0.31	7.96 ± 0.44	0.1306
	White adipose tissue	4.53 ± 0.34	3.33 ± 0.36	0.0254*
*Relative weight (g/100 g)*				
	Liver	3.23 ± 0.07	3.09 ± 0.09	0.2242
	White adipose tissue	1.65 ± 0.10	1.27 ± 0.10	0.0189*

Values are mean ± S.E.M. (*n* = 10 per group). Unpaired Student’s *t*-test. Asterisks indicate statistically significant differences compared to the Control group. *p < 0.05

### Assessment of liver injury and lipid metabolism

**Fig. 1:**
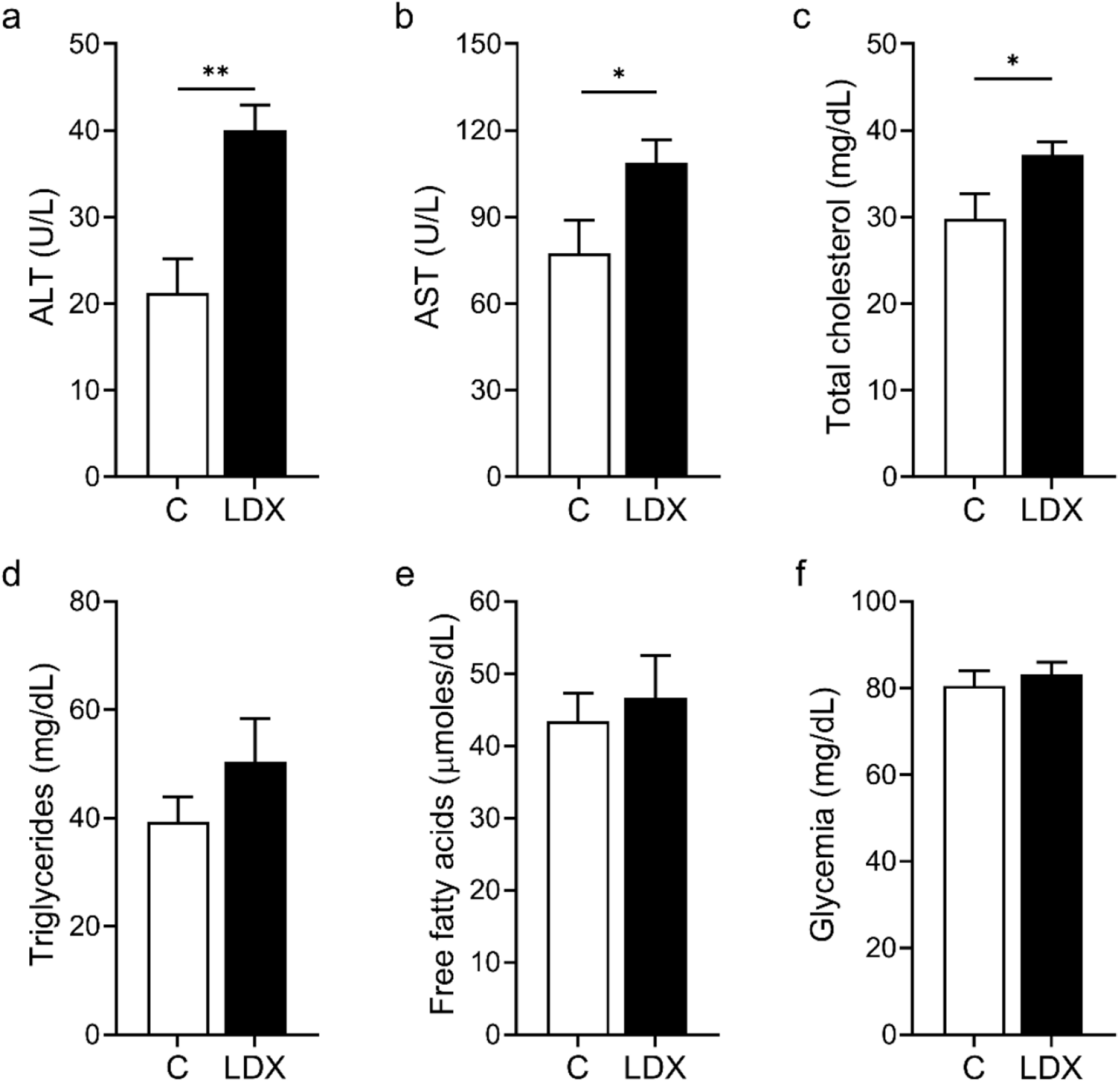
Plasma levels of hepatic transaminases (**a**, **b**), total cholesterol (**c**), triglycerides (**d**), free fatty acids (**e**), and blood glucose assessment (**f**). Data are mean ± S.E.M. (*n* = 10 per group). Unpaired Student’s *t*-test. Asterisks indicate statistically significant differences compared to the Control group. *p < 0.05; **p < 0.01. ALT, Alanine aminotransferase; AST, Aspartate aminotransferase

Plasma concentrations of ALT and AST, which are markers of liver injury, increased in animals that received LDX compared to the Control group (Fig. 1a and b).

In the assessment of lipid metabolism, no significant differences were observed in the plasma concentration of triglycerides (Fig. 1d), free fatty acids (Fig. 1e), and glycemia (Fig. 1f). However, the plasma concentration of total cholesterol was significantly higher in the animals that received LDX (Fig. 1c).

### Morphometric and histopathological analyses of the liver

Morphometric analysis (Table 2) showed that the dose of LDX used did not change the mean diameter of the main hepatic structures, which are the central vein, portal vein, hepatic artery, and bile duct. The average diameter of the sinusoid lumen near the main vessels was also not significantly different.

**Table 2:** Liver morphometric analysis

		Control group (C)	LDX group (LDX)	*P* value
*Mean diameter (µm)*				
	Central vein	50.6 ± 2.9	56.4 ± 2.6	0.1611
	Lumen of sinusoids near the central vein	5.57 ± 0.30	5.59 ± 0.37	0.9689
	Portal vein	33.3 ± 0.7	35.0 ± 2.2	0.4610
	Lumen of sinusoids near the portal vein	3.76 ± 0.24	3.73 ± 0.31	0.9366
	Hepatic artery	12.2 ± 0.5	11.5 ± 0.6	0.3913
	Bile duct	14.8 ± 1.0	15.0 ± 0.7	0.9233

Values are mean ± S.E.M. (*n* = 6 per group). Unpaired Student’s *t*-test. Asterisks indicate statistically significant differences compared to the Control group. *p* > 0.05

**Fig. 2:**
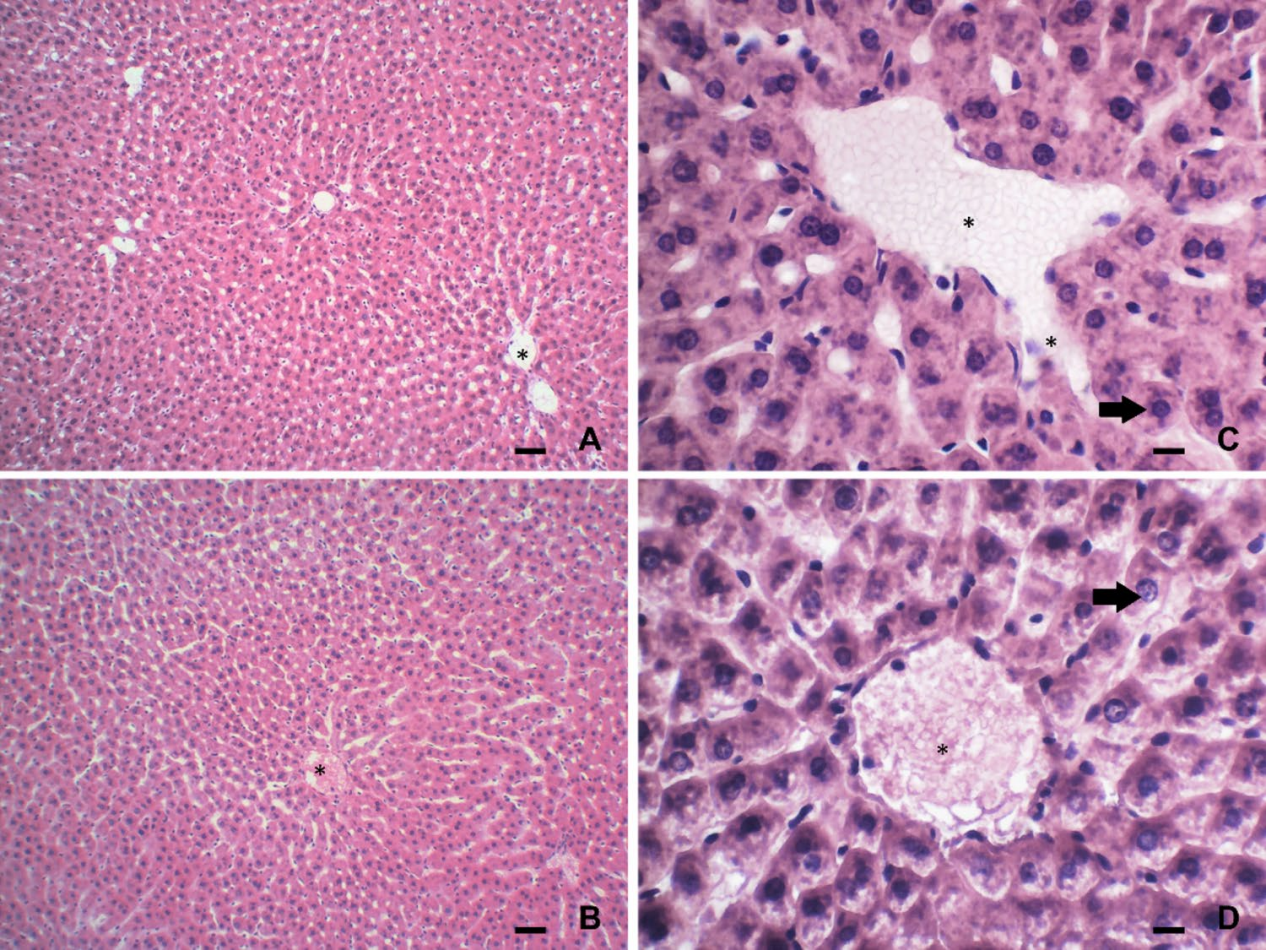
Photomicrograph of liver section from Control group (**A**, **C**) and LDX group (**B**, **D**). H&E, scale bar = 100 µm (**A**, **B**) and 10 µm (**C**, **D**). Asterisks denote typical vascular structures (e.g., central veins and sinusoids), and arrows indicate normal hepatocytes. These features highlight the preserved hepatic architecture and the complete absence of morphological alterations, such as necrosis or steatosis, in both groups

Histopathological analysis of the liver, as shown in Fig. 2, showed that sinusoidal and vascular congestion, foci of defense cells in the parenchyma, steatosis, and necrosis were not observed in either group.

### Liver inflammatory profile

With the toluidine blue technique, it was possible to count the number of mast cells in the extent of the cut, and differentiate them as intact or degranulated, but there was no difference between the Control and LDX groups (Table 3). Furthermore, in the HE stains, leukocytes adhered to the main vessels were counted and also demonstrated no significant difference between the groups (Table 3).

**Table 3:** Number of leukocytes adhered to the vessel lumen and mast cell count per section

		Control group (C)	LDX group (LDX)	*P* value
*Leukocytes adhered*				
	Central vein	0.70 ± 0.12	0.97 ± 0.12	0.1449
	Portal vein	0.78 ± 0.19	0.83 ± 0.15	0.8398
*Mast cells*				
	Intact	1.17 ± 0.48	1.17 ± 0.31	> 0.9999
	Degranulated	1.17 ± 0.31	0.50 ± 0.22	0.1099
	Total	1.17 ± 0.27	0.83 ± 0.21	0.3387

Values are mean ± S.E.M. (*n* = 6 per group). Unpaired Student’s *t*-test. Asterisks indicate statistically significant differences compared to the Control group. *p* > 0.05

**Fig. 3:**
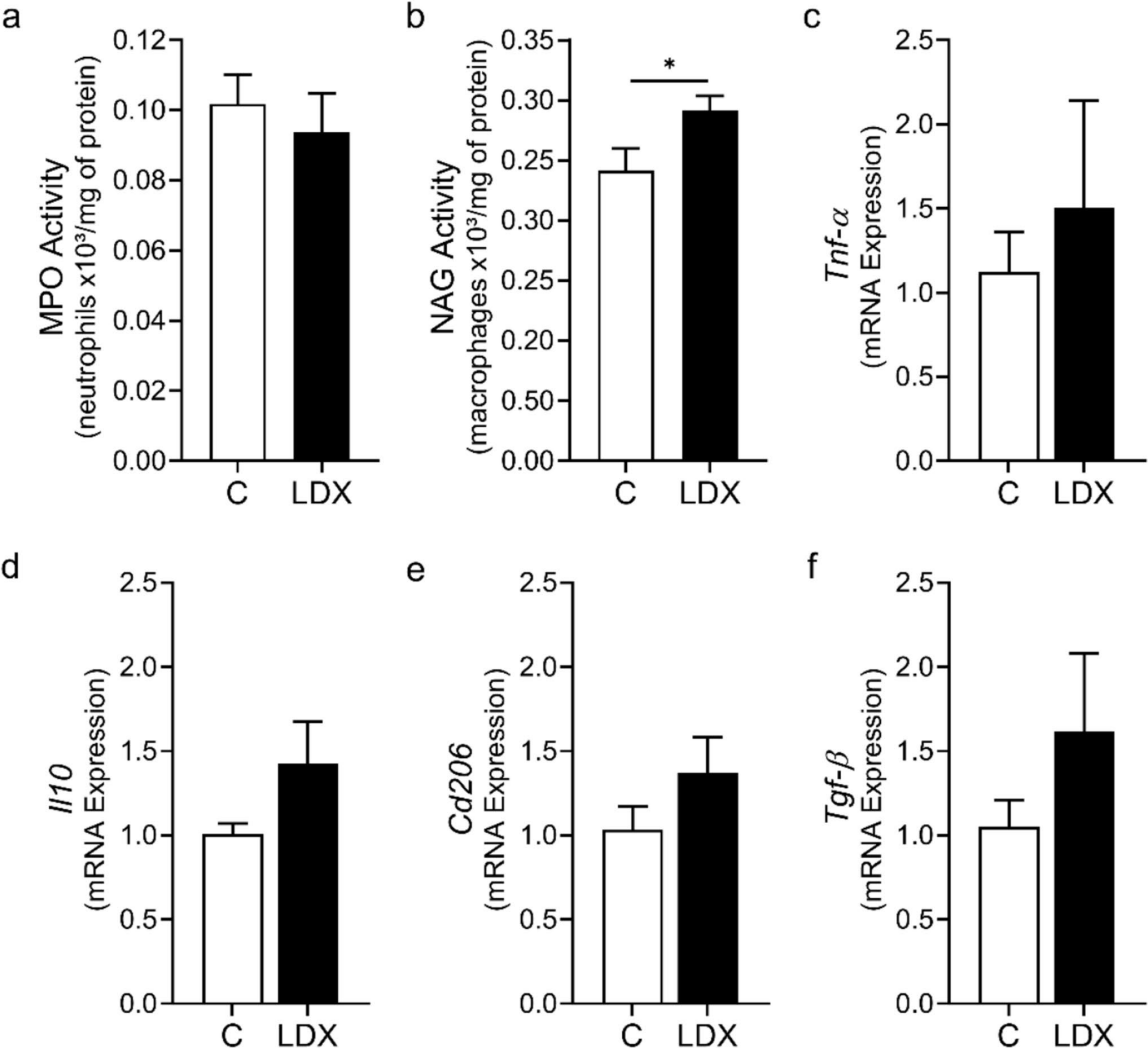
Hepatic inflammatory profile (**a**, **b**). Enzymatic activity of Myeloperoxidase—MPO (**a**) and N-acetyl-β-D-glucosaminidase—NAG (**b**). **c**–**f**. Gene expression analysis of *Tnf-α* (**c**), *Il10* (**d**), *Cd206* (**e**) and *Tgf-β* (**f**) by RT‐qPCR, normalized to *Gapdh*. Data are mean ± S.E.M. (*n* = 10 per group for **a** and **b**; *n* = 5–4 per group for **c**–**f**). Unpaired Student’s *t*-test. Asterisks indicate statistically significant differences compared to the Control group. *p < 0.05

**Fig. 4:**
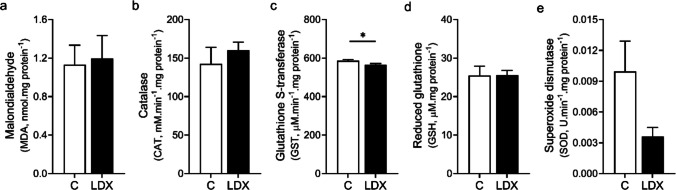
Oxidative status assay in the liver. Malondialdehyde concentration (**a**), Catalase (**b**), GST (**c**) and SOD activity (**e**), GSH quantification (**d**). Data are mean ± S.E.M. (*n* = 10 per group of graphs **a**, **b**, **c** and D; *n* = 9–7 per group on graph **e**). Unpaired Student’s *t*-test. Asterisks indicate statistically significant differences compared to the Control group. *p < 0.05

Figure 4 presents the results from the evaluation of MPO and NAG activity. There was no significant difference in MPO activity (Fig. 3a) between the LDX and Control groups, however it is possible to observe that LDX increased NAG activity in the liver (Fig. 3b). This indirectly indicates an increase in the recruitment of macrophages, but not neutrophils, in the liver.

Corroborating these data, we evaluated the gene expression of TNF (Fig. 3c), IL-10 (Fig. 3d), CD206 (Fig. 3e), and TGF-β (Fig. 3f) by RT-qPCR. None of these genes showed significantly altered expression in the LDX group when compared to the Control group.

### Hepatic oxidative status

LDX administration did not induce an increase in hepatic lipid peroxidation when assessing MDA concentration (Fig. 4a). Furthermore, when evaluating antioxidant enzymes, no significant differences were observed in CAT activity (Fig. 4b), GSH quantification (Fig. 4d), and SOD activity (Fig. 4e) between groups. However, there was a reduction in GST activity in the LDX group compared with the Control group (Fig. 4c).

### Histochemical analyses

**Fig. 5:**
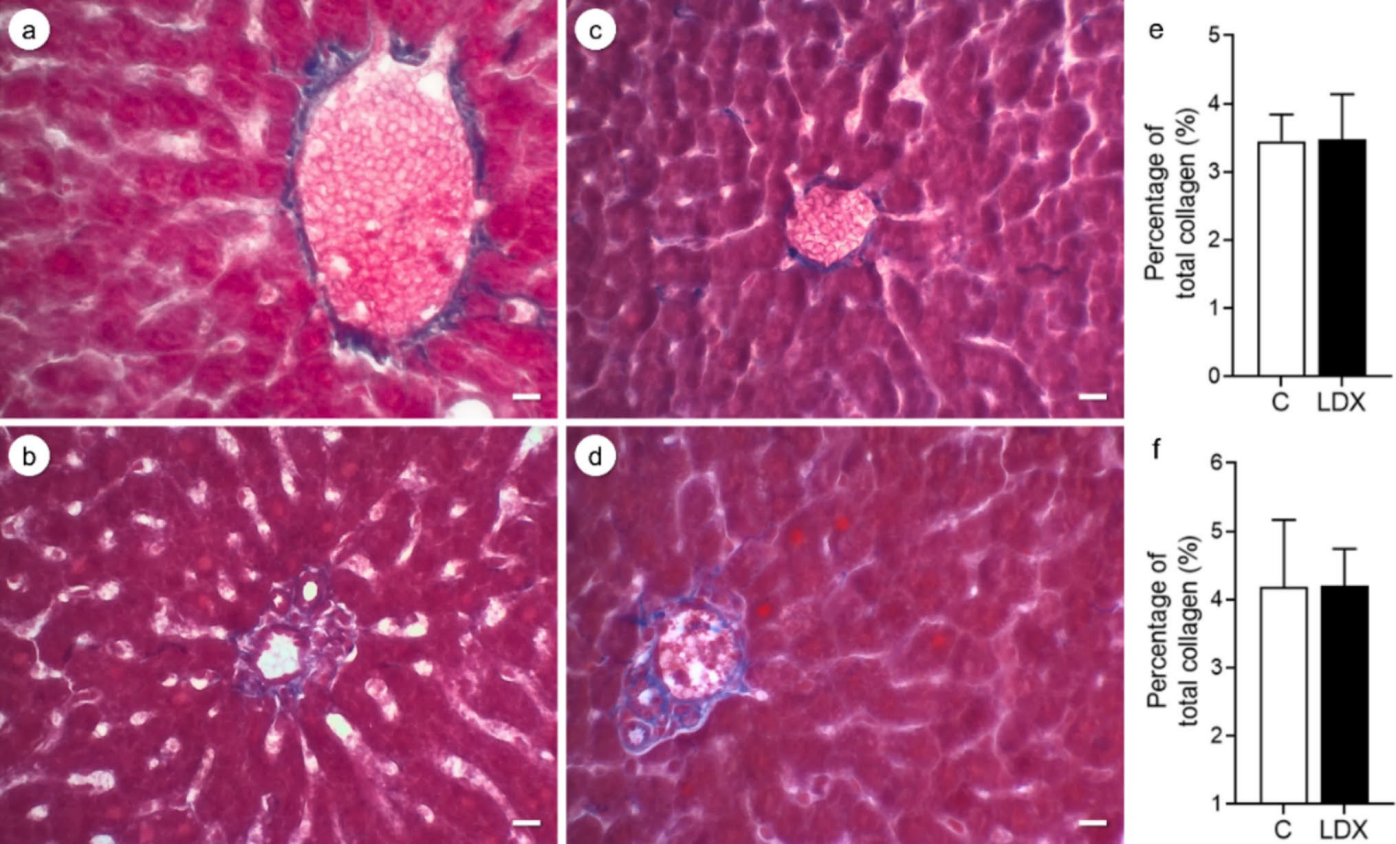
Photomicrographs of liver sections from the central region (**a**, **c**) and the portal region (**b, d**) staining total collagen in blue. (**a** and **b**) Control group; (**c** and **d**) LDX group. Percentage of total collagen in the central (**e**) and portal (**f**) regions. Azan Heidenhain trichrome staining. Scale bar: 10 µm. Data are mean ± S.E.M. (*n* = 6 per group). Unpaired Student’s *t*-test. Asterisks indicate statistically significant differences compared to the Control group. *p* > 0.05

**Fig. 6:**
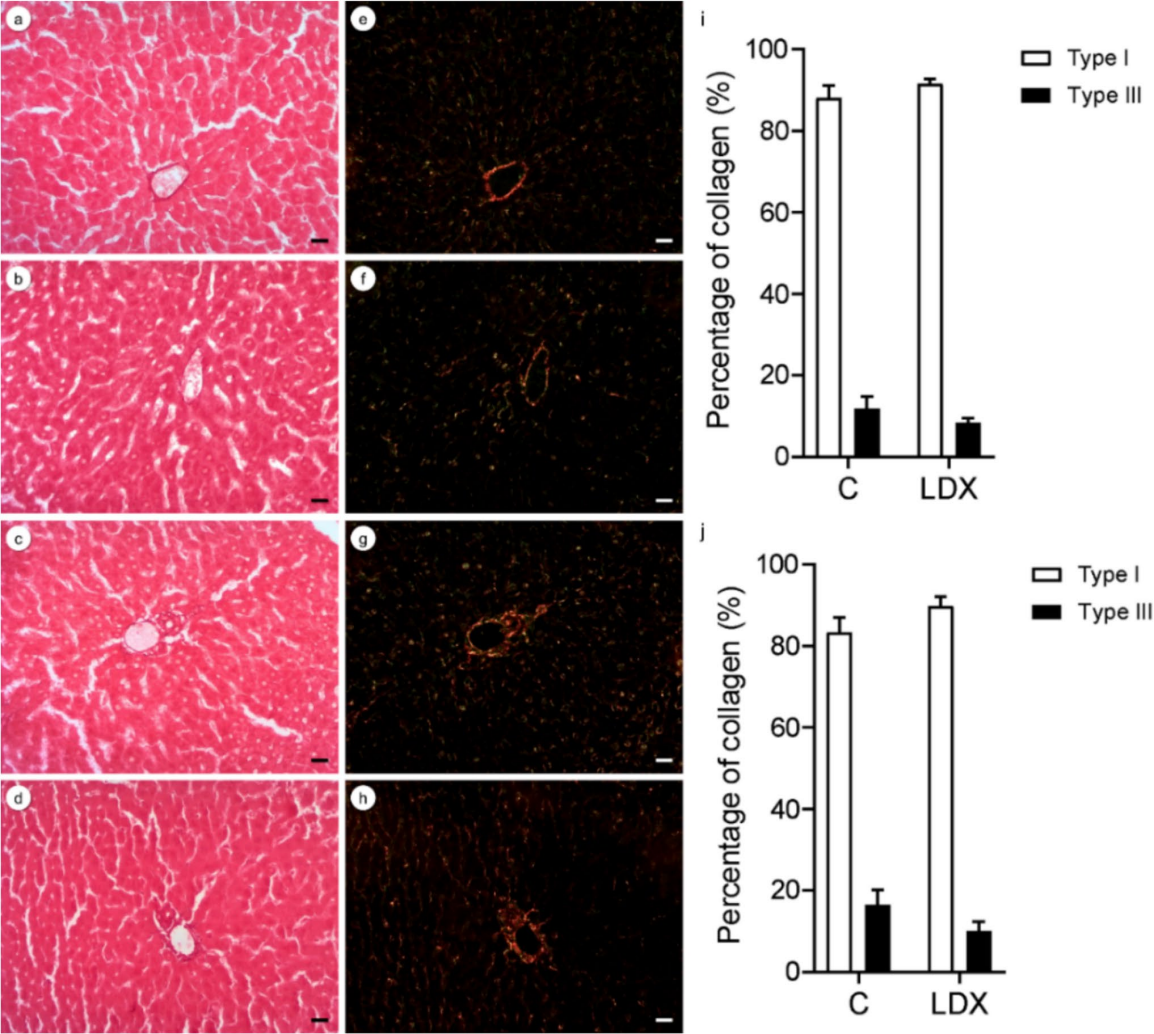
Collagen type I and III distribution in liver regions. Non-polarized (**a**–**d**) and polarized (**e**–**h**) photomicrographs of liver sections from the central (**a, b, e, f**) and portal regions (**c, d, g, h**). (**a, e, c, g**) Control group; (**b, f, d, h**) LDX group. Picrosirius red staining. Scale bar: 20 µm. Data are mean ± S.E.M. (n = 6 per group). Unpaired Student’s t test. Asterisks indicate statistically significant differences compared to the Control group. *p* > 0.05

The percentage of total collagen (Fig. 5) and of the different types of collagens (Fig. 6) evidenced that there were no significant differences between the experimental groups for these parameters.

## Discussion

In the present study, we showed that exposure to LDX during the peripubertal period alters hepatic parameters in pubertal male rats, and that immune and antioxidant systems may be involved in modulating these effects.

Interestingly, it was demonstrated that LDX at the dose used reduces white adipose tissue in male Wistar rats, without significantly altering weight gain. Corroborating our findings, Krishnan and Montcrief (2007) reported that after 28 days of oral LDX treatment (20 mg/kg/day), Sprague–Dawley male rats aged approximately 7 weeks showed minimal toxicological effects and no change in body weight parameters. However, the same authors also noted that rats receiving different doses of LDX (40 and 80 mg/kg/day) exhibited a reduction in mean body weight and weight gain. In contrast, Ekstrand et al. (2019) observed a significant decrease in mesenteric, renal, and epididymal adiposity, along with a reduction in body weight in male Long-Evans rats (approximately 5 weeks of age at the start of the study) after 1.5 mg/kg LDX oral administration for 20 days and a further 9 days of withdrawal period. Together, these data suggest that alterations in body parameters are highly dependent on LDX dose and treatment duration. In our model, a low dose (11.3 mg/kg) over approximately 5 weeks was sufficient to reduce adipose tissue without restricting overall body weight in male rats.

Alongside the adipose tissue reduction, we observed an increase in ALT and AST after LDX administration. The plasma levels of these transaminases is a classic and reliable marker of liver injury (Sookoian and Pirola 2015), as hepatocellular damage directly causes the leakage of these intracellular molecules into the systemic circulation (Reichling and Kaplan 1988; Kamiike et al. 1989). However, in the present study, despite the increase in ALT and AST levels, no morphological alterations were found in the liver. In this context, the findings suggest that LDX exposure induces early or subclinical liver injury, characterized by increased membrane permeability or reversible cellular stress rather than necrosis or tissue disorganization. This interpretation is consistent with toxicological models in which biochemical markers and oxidative status precede histopathological changes, particularly under conditions of mild or moderate exposure, or adaptation after exposure (Church and Watkins 2017; Ye et al. 2018; McGill and Jaeschke 2019).

When the liver is affected by disease or injury, lipid metabolism can be altered (Liangpunsakul and Chalasani 2019; Willis et al. 2021). As the central organ regulating lipid homeostasis, the liver plays a key role in cholesterol metabolism (Wang et al. 2018), while white adipose tissue serves as a major reservoir of free cholesterol, contributing to the balance between tissue storage and circulating cholesterol levels (Krause and Hartman 1984; Zhang et al. 2019). Although we found a significant elevation in plasma total cholesterol following peripubertal LDX administration, the lack of a detailed systemic lipid profile is a limitation of the present study. We did not quantitatively evaluate specific circulating lipoprotein fractions (e.g., HDL, LDL, and VLDL fractions). Because the liver governs cholesterol homeostasis and lipoprotein assembly, identifying which specific fraction drives this hypercholesterolemia is essential to fully understand the metabolic impact of the drug (Yamauchi et al. 2026). Future investigations evaluating these parameters may help clarify the metabolic impact of LDX on hepatic function.

Furthermore, based on the inflammatory profile evaluations, the increase in NAG activity indirectly indicated an increase in macrophages in the liver after exposure to LDX. Although macrophages are highly heterogeneous, they are often broadly categorized into two main subsets, M1 and M2, based on their inflammatory profiles (Shapouri‐Moghaddam et al. 2018). M1 macrophages are known to be pro-inflammatory (Bashir et al. 2016), and M2 macrophages are anti-inflammatory, with pro-resolving characteristics, repairing or healing wounds (Wang et al. 2019). Macrophages are essential for hepatic regeneration, as their depletion impairs tissue remodeling and metabolic recovery following liver injury (You et al. 2013; Miura et al. 2021). The predominance of macrophage-associated activity, together with unchanged neutrophil activity, is consistent with inflammation resolution or tissue adaptation (Medzhitov 2008; Zigmond et al. 2014), which may explain the increased NAG activity without overt inflammatory activation.

Notably, the absence of changes in TNF-α, TGF-β, IL-10 and CD206 expression suggests that macrophage recruitment induced by LDX does not correspond to classical M1 or M2 polarization states. Increasing evidence indicates that the M1/M2 dichotomy represents an oversimplified model, as tissue macrophages frequently adopt intermediate or homeostatic activation states depending on local metabolic cues. In this context, macrophage accumulation may reflect a surveillance or adaptive response to mild hepatic stress rather than canonical inflammatory or fibrogenic activation (Wen et al. 2021; Peng et al. 2025).

A limitation of the present study is that inflammatory mediators were evaluated at the transcriptional level using RT-qPCR, without confirmation at the protein level. Since mRNA expression does not always correlate with protein abundance or biological activity, subtle changes in cytokine signaling or macrophage functional states cannot be completely excluded.

Liver injuries can cause redox imbalance, resulting in oxidative stress (Zhu et al. 2012). Inflammatory response and drug-induced toxicity are examples of increased production of reactive oxygen species (ROS), since the liver has high metabolic and detoxification activity (Andrade et al. 2015).

To date, no studies have evaluated the hepatic oxidative status following continuous LDX exposure. However, D-AMPH has been associated with hepatotoxic effects (Carvalho et al. 2001a; El-Tawil et al. 2011). In the present study, conventional oxidative damage markers were largely unchanged, although GST activity was reduced, suggesting an early impairment in detoxification capacity rather than widespread oxidative injury (Faustino et al. 2011).

The mechanisms underlying LDX-induced hepatic alterations may involve catecholamine-mediated toxicity and mitochondrial dysfunction, processes previously associated with amphetamine exposure. LDX is metabolized to D-AMPH, which increases catecholaminergic signaling and may promote oxidative metabolism and ROS generation, particularly in highly metabolic organs such as the liver (Carvalho et al. 1997, 2001b; El-Tawil et al. 2011). Excess catecholamines may impair hepatic mitochondrial function through monoamine oxidase–dependent oxidative metabolism, promoting electron transport chain dysfunction and increasing mitochondrial ROS production, ultimately leading to early disturbances in redox homeostasis and hepatocellular injury (Shehu et al. 2017; Lelou et al. 2022). In the present study, the absence of consistent changes in classical oxidative damage markers, together with reduced GST activity, suggests an initial impairment in detoxification capacity. Additional analyses, including markers of protein and DNA oxidation, lipid peroxidation products, and the evaluation of mitochondrial-dependent antioxidant pathways, would further clarify the redox mechanisms involved.

In summary, our data indicate that a long-term dose of LDX induces early subclinical hepatic stress in pubertal rats, as evidenced by increased ALT and AST levels. Concurrently, macrophages are recruited for silent tissue repair, accompanied by reductions in adipose tissue weight without significant changes in body weight gain. We conclude that peripubertal LDX administration in male rats triggers early, reversible hepatocellular stress and a subtle redox imbalance, reinforcing the need for hepatic monitoring during continuous psychostimulant use in pubertal age.

## Data Availability

The data that support the findings of this study are available from the corresponding author, F.G.A., upon reasonable request.
